# Milk Yield, Major Milk Components and Macro Minerals in Blood Serum of Lactating Donkeys, as Affected by Dietary Trace Element Supplementation and Stage of Lactation

**DOI:** 10.3390/ani15081073

**Published:** 2025-04-08

**Authors:** Francesco Fantuz, Luca Todini, Elisabetta Salimei, Antonella Fatica, Pierluigi Mariani, Fausto Marcantoni, Stefano Ferraro

**Affiliations:** 1 Scuola di Bioscienze e Medicina Veterinaria, Università degli Studi di Camerino, Via Gentile III da Varano, 62032 Camerino, Italy; francesco.fantuz@unicam.it (F.F.); luca.todini@unicam.it (L.T.); pluigimariani@gmail.com (P.M.); 2 Dipartimento Agricoltura, Ambiente e Alimenti, Università degli Studi del Molise, Via Francesco de Sanctis 1, 86100 Campobasso, Italy; salimei@unimol.it; 3 Scuola di Scienze e Tecnologie, Università degli Studi di Camerino, Via Madonna delle Carceri snc, 62032 Camerino, Italy; fausto.marcantoni@unicam.it (F.M.); stefano.ferraro@unicam.it (S.F.)

**Keywords:** dairy donkey, dietary minerals, stage of lactation, blood, milk yield, milk composition

## Abstract

The aim of this experiment was to test the effects of both dietary trace element supplementation and the stage of lactation on the milk yield per milking and the major milk components, as well as the blood concentrations of total Ca, P, Mg, Na, and K in lactating donkeys. Over a 3-month period, 16 clinically healthy lactating donkeys were used to collect milk and blood samples and to evaluate the milk yield per milking. The experimental subjects were randomly divided into two homogeneous groups, control (CTL) and trace element (TE). The donkeys in the TE group were fed a diet supplemented with Zn, Fe, Cu, Mn, Se, Co, and I. The dietary treatment did not significantly affect neither the milk yield per milking nor the milk macro ingredients. However, these variables were significantly affected by the stage of lactation. The serum concentrations of total Ca, P, Na, and K were not affected by the dietary treatment, but the serum Mg level in the TE group was significantly higher than that in the CTL group (26.6 vs. 25.0 mg/L). The effect of stage of lactation was found to be significant for all the investigated minerals in the blood serum, and negative trends were observed for serum Ca, P, Mg, Na, and K during the experimental period.

## Simple Summary

In recent years, donkey farming has gained increasing importance, particularly for milk production intended for human consumption, but the nutrient requirements for lactating donkeys are not yet well defined. Furthermore, only few data are available in the literature on macro minerals in the blood serum of lactating donkeys. As part of a larger study, this experiment aimed to test the effects of both dietary trace element supplementation and the stage of lactation on milk yield per milking and the major milk components and on the concentrations of total Ca, P, Mg, Na, and K in the blood serum of lactating donkeys. Dietary supplementation with essential trace elements did not significantly affect the milk yield per milking nor the major milk components. The concentrations of total Ca, P, Na and K in the blood serum were not affected by dietary trace element intake, except for Mg, whose concentration increased in the supplemented animals. The stage of lactation significantly affected the milk yield per milking and its composition and all the investigated elements in the blood serum. These findings are of interest both for clinical practice and research, as well as for donkey farming and feeding.

## 1. Introduction

Donkeys, or asses (class Mammalia, order Perissodactyla, family Equidae, genus *Equus*, species *asinus*), are reported to have been domesticated in Africa about 5000 years ago [1]. The species has historically been valued for its adaptability and versatility, serving as essential animals for transportation and agricultural work in diverse environmental and farming conditions worldwide. As a result of the mechanization of transportation and agriculture during the 20th century, the asinine population suffered a substantial decline in many industrialized countries, despite maintaining or gaining importance for rural economies in semiarid and mountainous areas for agricultural tasks and transportation [1]. Overall, the donkey population worldwide increased from approximately 47 to 53 million heads from 2012 to 2023 due to the increase in Africa (from 25 to 34 million), despite the decrease in Asia (from 15 to 12 million) [2].

In recent years, donkey farming has also gained increasing importance for milk production, as this milk has been used in human nutrition since ancient times and was traditionally used in Europe as a human milk substitute [3]. Clinical studies have shown that due to its similarities with human milk, donkey milk can be used when adequately implemented in the diets of children with an IgE- and non-IgE-mediated cow milk allergy [4]. Moreover, donkey milk has gained interest for adults with inflammatory or allergic ailments, as well as for healthy elderly people [3,5]. Knowledge about donkey milk composition has greatly improved in recent years. When compared to cow milk, donkey milk is considered similar to human milk due to its relatively low protein (and casein) and high lactose contents. The ash content in donkey milk is also more similar to that in human milk than that in cow milk [3,5,6]. The fat content is highly variable and generally low, being mainly related to the efficiency of milk removal from the mammary gland [7]. Currently, donkey milk is mainly marketed in the food sector as raw, pasteurized, or powdered (either spray- or freeze-dried), and as fermented derivatives. Additionally, it is increasingly used in the non-food sector as an ingredient for cosmetic formulations [5,6,8].

Appropriate feeding is critical for maintaining animal health and supporting the metabolic processes required for lactation. However, despite the growing importance of donkey farming, the nutrient requirements during lactation are not yet well defined for the dairy donkey, and the number of studies on the nutritional management of lactating donkeys remains limited, particularly with regard to mineral nutrition [8,9,10,11]. Dietary essential minerals, classified as macro minerals (Ca, P, Mg, Na, K, Mg, and S) and trace elements (Zn, Fe, Cu, Mg, Se, I, Co, and Mo) based on their concentration in animal tissues and in the diet, play a primary role in all biological processes due to their four major functions, including structural, physiological, catalytic, and hormonal or regulatory. Essential macro minerals and trace elements support general health, reproduction, optimal growth, the immune system, and productivity, so a dietary mineral imbalance will induce negative effects in an animal [12,13,14].

The assessment of the blood biochemical profile, which often includes the analysis of essential macro minerals and trace elements, is important in both clinical practice and research for the determination of an animal’s physiological or pathological status. The data available in the literature regarding the blood serum macro minerals in donkeys are not abundant and mainly refer, as influencing factors, to the effects of age, gender, breed, and physiological status, with a focus on pregnancy. The few data existing on lactating donkeys are limited to early lactation [15,16,17]. On the other hand, the minerals in blood are generally subjected to homeostatic control [13], and blood serum analysis may have limited sensitivity for the detection of an altered mineral status related to dietary changes [18]. Nevertheless, according to Schweinzer et al. [14], the oversupply of K and Mo in sheep and Fe in goats and the undersupply of Zn in sheep and goats could be detected by blood serum analysis.

The aim of this experiment was to study the effects of both dietary trace element supplementation and the stage of lactation on the milk yield per milking and the major milk components and on the total Ca, P, Mg, Na and K concentrations in the blood serum of lactating donkeys.

## 2. Materials and Methods

### 2.1. Animals, Diets, and Sampling

During a 3-month period, 16 clinically healthy lactating donkeys (Martina Franca-derived population; from 32 to 58 d after foaling; average body weight 205 kg; from 6 to 12 yr old; and from 3 to 7 parities at the beginning of the trial) were used to provide milk and blood samples. In this field study, the donkeys were reared at a commercial dairy donkey farm, where dairy animals are machine-milked twice a day (11:00 and 16:00). As a part of a larger study carried out in 2009 focused on the essential trace elements in donkey milk, the experimental subjects were randomly divided into two homogeneous groups: control (CTL) and trace element (TE) groups. The donkeys were uniformly distributed across the groups according to their initial days of lactation and body weight. The donkeys in each group had free access to water and meadow hay (as fed: DM 88.2 g/100 g; CP 12.2 g/100 g; NDF 53.5 g/100 g) and were group-fed 2.5 kg of pelleted mixed feed (as fed: DM 88.5 g/100 g; CP 12.6 g/100 g; NDF 25.9 g/100 g) divided in two meals per day. The mixed feed for the TE group had the same composition as that for the CTL, but it was supplemented with a commercial trace element premix, providing 185 mg of Fe (ferrous carbonate), 36 mg of Cu (copper sulfate), 163 mg of Zn (zinc oxide), 216 mg of Mn (manganese oxide), 3.20 mg of I (calcium iodate), 2.78 mg of Co (cobalt sulfate), and 0.67 mg of Se (sodium selenite) per kg of mixed feed. Both the groups were fed the control diet two weeks before the beginning of sampling, and the supplemented mixed feed was gradually administered to the animals in the TE group starting at the 1st sampling period. Samples of hay and mixed feeds were collected and analyzed for element concentrations by inductively coupled plasma-MS (ICP-MS; 7500cx series; Agilent Technologies, Santa Clara, CA, USA) [19]. The concentration of Na in hay and in the CTL and TE mixed feeds were, respectively, 0.015, 0.60, and 0.60 g/100 g (as fed). The concentration of K in hay and in the CTL and TE mixed feeds were, respectively, 2.84, 1.05, and 1.06 g/100 g (as fed). The calculated concentrations of Ca, P, K, Na, and Mg of the CTL and TE diets (assuming a daily intake of 5.0 and 2.5 kg, respectively, of hay and mixed feed) [19] were the same for both the groups, as follows: 0.90 g Ca, 0.36 g P, 2.25 g K, 0.21 g Na, and 0.23 g/100 g Mg (as fed). The mineral requirements of lactating donkeys are not defined; however, the intake of the investigated macro minerals and trace elements calculated for the donkeys in the CTL group would meet or exceed the mineral dietary requirements of lactating mares (200 kg BW), except for Se [20].

Dams were housed with foals that were separated from the dam 3 h before milking. Every 2 weeks, individual milk samples were collected by mechanical milking [21] during the morning milking, and the milk yield was recorded. Aliquots of milk samples were refrigerated until the analyses of protein, fat, and lactose contents, occurred within 24 h from collection. Additional aliquots were stored at −21 °C until ash content analysis. Blood samples were collected every 2 weeks, just after morning milking, by jugular venipuncture using evacuated tubes (Venoject, Terumo Europe NV, Leuven, Belgium) without anticoagulant. The samples were left to clot, and serum aliquots were stored at −21 °C until the analysis of minerals.

### 2.2. Blood Serum and Milk Analyses

All solutions were prepared using ultrapure water obtained from a Millipore Milli-Q system (resistivity 18.2 MΩ cm). For element analysis, 0.5 mL of thawed blood serum samples (n = 112) was diluted 1:20 with an HNO_3_ solution (1%) [19], and the concentrations of total Ca, P, Mg, Na, and K in acidified blood serum were measured by inductively coupled plasma-mass spectrometry (Agilent Technologies, 7500 cx series, Santa Clara, CA, USA) The operating conditions were as previously described [19]. Calibration curves for investigated elements were obtained using aqueous (1% nitric acid) standard solutions prepared with appropriate dilution of stock standards (Fluka Analytical, Aldrich, Milan, Italy). The accuracy of the analytical procedure was checked within each batch analysis, including blank samples and the certified reference material, Seronorm Trace Elements Serum L-2 (Sero As, Billingstad, Norway). The recovery of certified elements was in good agreement (from 92 to 96%) with the certified (Ca, P, and Mg) or approximate (K and Na) values.

Milk samples were analyzed using infrared (Milkoscan 605, Foss Italia, Padova, Italy) for protein, fat, and lactose contents, and ash content was measured gravimetrically [21].

In the current study, the blood serum trace element concentrations, the milk macro mineral and trace element concentrations, the blood plasma and milk thyroid hormones concentrations, and the feedstuff chemical composition were as previously published [19,22,23,24,25].

### 2.3. Statistical Analysis

Statistical analyses were performed using SPSS Statistic Data Editor version 25 (SPSS Inc., Chicago, IL, USA), and individual animals served as experimental unit. The variables were checked for normal distribution using the Kolmogoroff–Smirnoff test, and in cases of non-normality, as for milk yield per milking, milk fat, and serum Na and K, the data were logarithmic-transformed. The data were processed by ANOVA for repeated measures to evaluate the effects of the dietary treatment and of the stage of lactation. Least squares means were calculated using the following model:

y_ijk_ = μ + α_i_ + β_ij_ + γ_k_ + (αγ)_ik_ + ε_ijk_,

where y = dependent variable; μ = overall mean; α = treatment (i = 1 to 2); β = animal effect within the treatment (j = 1 to 8); γ = sampling time (k = 1 to 6); αγ = treatment × sampling time interaction; and ε = error. In the case of significant effects (*p* < 0.05), the differences between means were analyzed by least significant difference. The data from the 1st sampling were used as covariate when significant (milk yield per milking, and serum P and K, and Na-to-K ratio).

## 3. Results

### 3.1. Effect of Dietary Trace Elements Supplementation on Milk Yield per Milking, Milk Gross Composition, and Serum Macro Minerals

The dietary supplementation with trace elements did not significantly affect the milk yield per milking nor the major milk components (Table 1). The overall means (±SEM) for milk yield per milking, protein, fat, lactose, and ash contents were 555.1 (±20.6) mL/milking, 1.74 (±0.02) g/100 g, 0.22 (±0.05) g/100 g, 7,07 (±0.02) g/100 g, and 0.39 (±0.01) g/100 g, respectively.

The blood serum concentrations of Ca, P, K, and Na were not affected by the dietary treatment, but the serum from the trace-element-supplemented donkeys contained higher (*p* < 0.05) concentrations of Mg compared to those of the animals in the CTL group (Table 2). The overall means (±SEM) blood serum concentrations of total Ca, P, Mg, Na, and K in the lactating donkeys were, respectively, 129.6 (0.64) mg/L, 103.9 (2.26) mg/L, 25.8 (0.33) mg/L, 137.1 (0.34) mmol/L, and 4.65 (0.04) mmol/L, and the Na/K ratio was 29.6 (0.31).

### 3.2. Effect of Stage of Lactation

**Figure 1:**
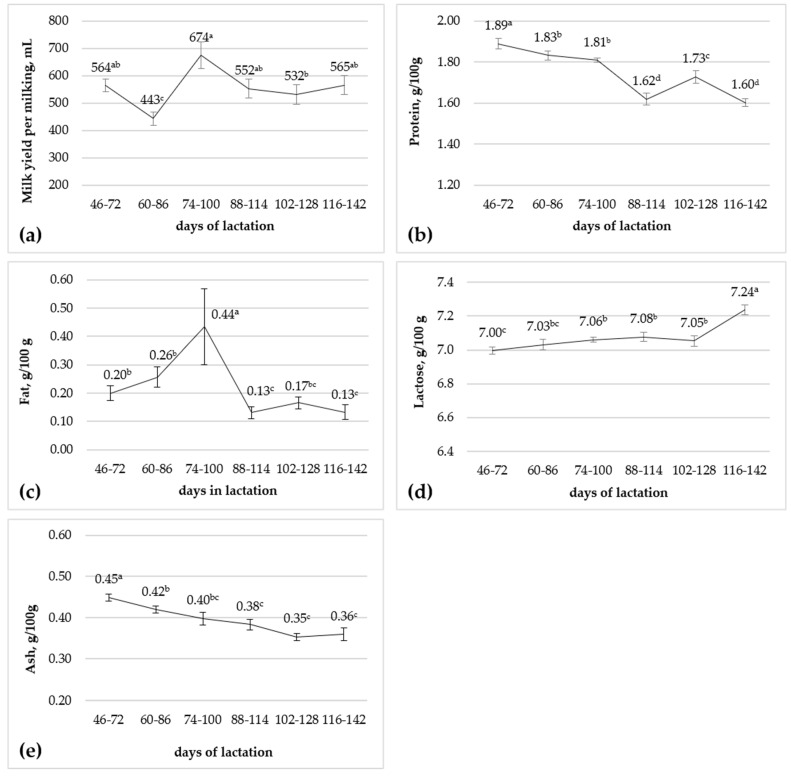
Effect of stage of lactation on milk yield per milking (**a**) and milk protein (**b**), fat (**c**), lactose (**d**), and ash (**e**) contents (n = 16). ^a–d^ Means with different superscripts differ (*p* < 0.05). Bars are standard error of mean.

The effect of the stage of lactation was significant (*p* < 0.01) for the milk yield per milking and for the investigated major milk components (Figure 1). The milk yield per milking and the fat content changes followed an inconsistent trend, whereas the protein and ash contents followed a decreasing trend during the considered lactation period. The milk concentration of lactose was constant during the intermediate experimental period, with significantly lower and higher values, respectively, at the first and at the last sampling times (Figure 1).

**Figure 2:**
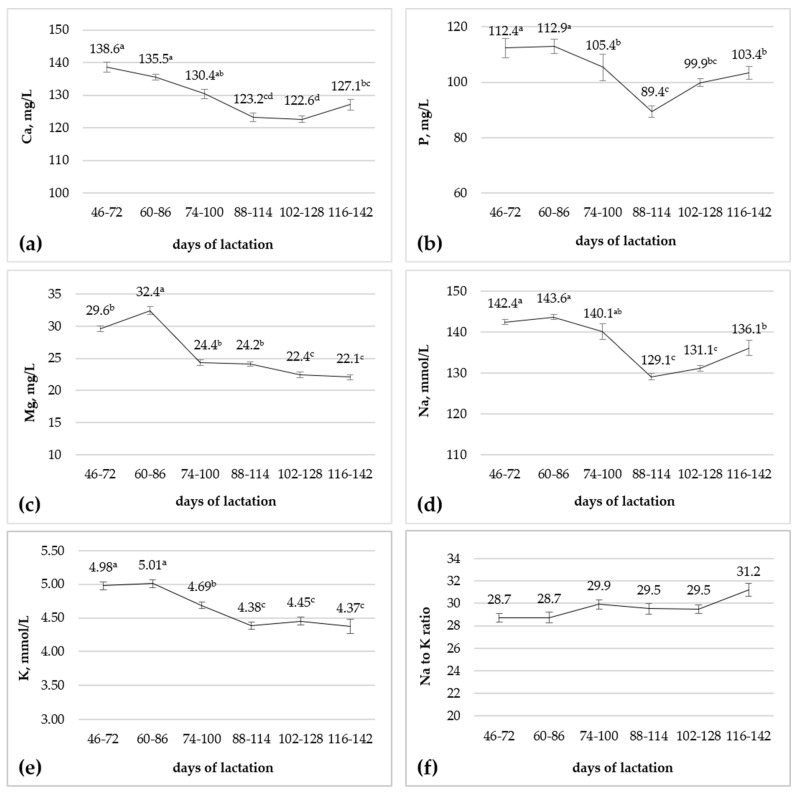
Effect of stage of lactation on Ca (**a**), P (**b**), Mg, (**c**), Na (**d**), and K (**e**) concentrations and on Na-to-K ratio (**f**) in blood serum of lactating donkeys (n = 16). ^a–d^ Means with different superscripts differ (*p* < 0.05). Bars are standard error of mean.

**Figure 3:**
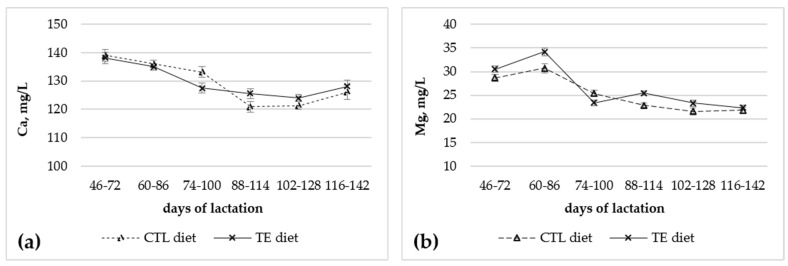
Changes in serum Ca (**a**) and Mg (**b**) levels during lactation in donkeys supplemented (TE, n = 8) or not (CTL, n = 8) with dietary trace elements. Bars are standard error of mean.

The effect of the stage of lactation was significant (*p* < 0.01) for all the investigated elements in the blood serum, but not for the Na:K ratio (Figure 2). Negative trends were observed for the blood serum Ca, P, Mg, Na, and K concentrations (Figure 2). The effect of the treatment x stage of lactation interaction was significant (*p* < 0.05) for serum Mg and Ca (Figure 3).

## 4. Discussion

In this experiment, we studied the milk yield per milking, the major milk components, and the macro minerals in the blood serum of lactating donkeys, taking into account the effects of dietary trace element supplementation and the stage of lactation.

The observed overall average milk yield per milking was similar or lower than the values reported in our previous studies [21,26,27,28,29] and fell in the wide range reported in the literature (from approximately 200 to 900 mL per milking) [8]. The variability in the literature data is the result of many influencing factors, such as the management of milking sessions, the milking procedure, the stage of lactation, individual milkability, body size and condition, genetics, and feeding [2,7,8,30,31]. The overall content of major milk components observed in our study is consistent with the typical donkey milk gross composition, confirming that, except for the low fat content, donkey milk is more similar to human milk in terms of protein, lactose, and ash contents than ruminant milk [3,5,6,32].

In donkeys, as in other species, total blood serum Ca and Mg are present in milk as free ions, bound to proteins (mainly albumin) or weakly associated with other molecules, such as bicarbonate, citrate, or phosphate [33,34]. Due to its instability, P is always bound to oxygen to form ion phosphate (PO_4_^3−^), which is largely found in an inorganic form in blood serum as free ions or complexed with small cations. Organic phosphate binds to proteins, lipids, and nucleic acids, among other molecules [34]. The data available in the literature on macro minerals in donkey blood serum are usually obtained by automated biochemical analyzers, which measure the concentrations of total Ca and Mg and that of inorganic P by colorimetric methods. Sodium and potassium are almost totally present in free ionic form in blood serum and are usually measured using an ion-selective electrode [35,36,37,38,39].

The concentrations of total serum Ca, K, and Na and of the Na/K ratio in our study fell in the reference ranges previously reported by Burden et al. [35] using 140 adult donkeys (29 females and 111 gelding males) in the UK, are slightly higher than the results reported by Goodrich and Webb [36] using 117 adult donkeys (females, intact males, and gelding males) from different locations across the USA, and lower than those by Trimboli et al. [37] using 78 adult Martina Franca breed donkeys (64 females and 17 males) in Italy. Furthermore, our results on Ca, Na, and K are substantially consistent with those reported for pregnant [38] and adult donkeys of different breeds used for different purposes and reared in different management systems and climatic conditions [39,40,41,42].

Fewer studies have examined the Mg concentrations in donkey serum. Our results fell within the reference range for adult donkeys reported by Goodrich and Webb [36], but slightly exceed the upper limit reported by Trimboli et al. [37]. Similar results have previously been reported by others [33,40,42], but much a lower serum Mg concentration (9.0 mg/L) was observed by Caldin et al. [39].

Regarding the macro minerals in the blood serum of lactating donkeys, in comparison with our results, similar Na concentrations and slightly lower average plasma Ca and K contents were reported for DeZhou donkeys breed during the first two weeks after foaling [17] and for the Amiata breed during the first eight weeks of lactation [15]. In Liaoxi donkeys 30 days after foaling, the average blood serum Mg level was found (38.4 mg/L) to be approximately 50% higher than our results, whereas the Na and K contents were slightly lower, and Ca was similar [16]. In the latter experiment, the concentration of Mg in the diet was almost ten times higher than that in the current study.

The published data for total P in donkeys are scanty. The plasma concentration of total P measured by inductively coupled plasma-optical emission spectrometry (ICP-OES) averaged approximately 95 mg/kg [17], similar to the current experiment. The average total Mg level in the same study was similar to our result, whereas those of Ca and K were lower [17]. In horses, the average total P level measured by ICP-OES was found to be approximately 75 mg/L for the plasma of nursing mares within 150 days of lactation [43] and approximately 150 mg/L for the serum of horses (6 females and 6 males) with age-related dependent differences [18].

The current study found that increasing the trace elements concentration in the diet of lactating donkeys did not have any effect on milk yield and composition. Positive effects of diet on donkey milk yield and composition were observed in a few reports. Supplementing the diet of late-gestating or lactating donkeys with different amounts of protein [44] or methionine [45] or substituting part of low-quality forage such as millet straw with forage of a better quality such as alfalfa [46] increased the milk yield per day and the milk protein content. To the authors’ knowledge, no published studies dealt with the effects of dietary trace elements on milk yield and composition in donkeys. In addition, the concentration of essential minerals in the diet is not always given in the available literature, and the specific dietary requirements of trace elements are not yet established. In dairy cows, increased milk yield, an improved reproductive performance, a decreased somatic cell count, decreased lameness and improved foot health, and decreased disease incidence are the most frequent positive effects reported after feeding a greater amount or more bioavailable chemical forms of essential trace elements [47]. Similarly to our experiment, Marchand et al. [48] observed that increasing the dietary concentrations of Co, Mn, and Zn over the recommendations had no significant effects on milk yield and on the milk concentration of protein, fat, and lactose in dairy cows.

In this study, we observed that supplementing the diet of lactating donkeys with trace elements did not affect the serum concentrations of total Ca, P, Na, and K, but that of Mg was increased in the treated animals. Our results on Ca, P, Na, and K are consistent with those on lactating mares. No significant effect was reported on the blood serum Ca level of lactating mares within 4 weeks after foaling when supplemented with Zn and Cu [49], nor on the plasma Mg, Ca, Na, and K levels of lactating mares supplemented with Cu during pregnancy within approximately 150 d after foaling [43]. Furthermore, in adult horses injected with 8 mg of Se and 240 mg of vitamin E, the serum concentration of Mg remained unchanged, and those of Ca and K decreased compared to those of the untreated animals [50]. In adult horses whose diet was supplemented with essential macro minerals (including Ca, P, Mg, Na, and Cl) and trace elements (including Zn, Cu, Mn, and I), the serum concentrations of total Ca, P, Mg, Na, and K did not change significantly compared to those of unsupplemented animals [18]. According to Hui et al. [16], different dietary energy levels affected the blood plasma concentrations of total Ca, P, Na, and K, but did not affect that of Mg in lactating donkeys.

As well as for Ca and P, extracellular Mg depends on intestinal absorption, bone exchange, and renal excretion [51,52]. The efficiency of the intestinal absorption of Mg depends on the balance between dietary Mg intake and requirements, being approximately at 50% at normal intake, but varying from 25 to 75% with high or low Mg intake, respectively [51,52]. In normal conditions, most of Mg is absorbed in the small intestine via a nonsaturable paracellular passive pathway, and a low proportion is absorbed in the large intestine via a saturable transcellular active pathway, mainly permitted by the transient receptor potential melastatin 6 (TRPM6) cationic channel [51,52,53]. However, serum Mg is primarily regulated by the kidneys in concert with the intestine, given that, once the requirement of Mg is fulfilled, the net kidney excretion of Mg is linearly related to the net intestinal absorption [51,53,54]. It is known that 10% of total body Mg is filtered daily by the kidney glomeruli [54], of which approximately 90–95% is reabsorbed, and only 5% is excreted in urine. Most of the filtered Mg is reabsorbed in the proximate tubule (about 10–20%) and in the thick ascending limb of the loop of Henle (about 70%) through a passive paracellular mechanism regulated largely by the modulation of tight junction permeability. The fine regulation of Mg reabsorption, and therefore of serum ionic Mg, occurs in the distal convoluted tubule, where approximately 10% of filtered Mg is reabsorbed through an active transcellular process controlled by TRPM6 [53,54,55]. Although found also in the large intestine, the epithelial Mg2+ channel TRPM6 in the distal convoluted tubule of the kidney is considered the major regulator of Mg^2+^ balance in the body [55]. The expression and activity of TRPM6 are reported to be affected by several hormones, including estrogen and insulin and epidermal growth factor, but there is no information about the possible effects of trace elements [55]. Data about serum ionic Mg are not available for this study, and we cannot speculate about the cause of the increased total serum Mg observed in trace-element-supplemented animals, also considering that total serum Mg may reflect changes in the serum albumin concentration [51,52]. It is worth noting that serum albumin also binds Ca, whose concentration remained stable in the present study.

We observed that the milk yield per milking and the major milk components changed significantly from about 30 to 150 days of lactation, but only the protein and ash contents showed a clear negative trend. The inconsistent trend that we have observed for the milk yield per milking is in agreement with the results of Salimei et al. [21], although a negative trend for milk yield was generally reported with the advancing of lactation [27,29,56,57,58,59,60,61,62]. Conversely, no significant differences on milk yield were reported from 30 to 300 days of lactation [63]. The declining trend observed during the experiment on milk protein content confirms the previous findings [21,56,57,60,62,63,64]. The low milk fat content followed an inconsistent trend, in agreement with the findings of Salimei et al. [21] and Malacarne et al. [60]. A constant or decreasing fat concentration during lactation was also reported [57,61,62,63,64]. Even though we have observed significant variations in the lactose content during lactation, the changes were of limited magnitude as usually reported [57,60,62,63,64]. The decline observed for milk ash content in the current experiment is consistent with the previous reports [3,57,60,63] and with the decrease in milk concentrations of Ca, P, and Mg observed within this experiment, as previously published [22]. The observed decrease in milk protein during lactation can be related to the decrease in the casein content reported by others [8,57,64], also considered that the majority of milk Ca (63%) and P (53%) and a relevant part of Mg (33%) are associated with the casein fraction in donkey milk [29]. However, the decrease in casein content was not confirmed in all the published studies [21,56,60,63]. The synthesis of milk components by mammary epithelial cells and the normal maintenance of lactation are regulated by galactopoietic hormones and growth factors (including prolactin, somatotropin, thyroid hormones, insulin, IGF-1, and glucocorticoids) and by the regular removal of milk [65], depending on regular suckling or the milking of the mammary gland, or both in our experiment. Among the mentioned factors, the milk concentration of triiodothyronine increased significantly in the treated animals within this experiment, but was not affected by the stage of lactation, whereas plasma triiodothyronine and thyroxine were not affected by the dietary treatment, but the levels increased during lactation, as previously published [24,25].

The blood serum concentration of the investigated elements significantly declined during lactation in this study, though remaining within the physiological range. The available data about the effect of the advancing of lactation on the blood mineral concentration in donkeys are scanty and limited to the first post-partum period. Decreasing trends for average plasma total Ca, P, Mg, and K during the first 14 days after foaling in donkeys have been observed by Hui et al. [17]. The plasma concentrations of Na and K in donkeys were reported to follow a decreasing trend also during eight weeks after foaling, whereas Ca remained unchanged [15]. In nursing mares, no remarkable changes were reported within 4 months of lactation for serum Ca, K, and Na [66], and the plasma Ca, Mg Na, and K concentrations did not change within 150 days of lactation [43]. Our results confirm the fact that the investigated macro minerals in blood are kept within a relatively constant range by homeostatic control [13], but physiological changes in their blood concentration occur, particularly during the physiological phases of high metabolic (and minerals) demands such as lactation.

## 5. Conclusions

The current results contribute to advances in the knowledge on the concentration of total Ca, P, Mg, Na, and K in the blood serum of female donkeys during mid lactation and add further data on donkey milk yield and composition. Under the current experimental conditions, dietary supplementation with essential trace elements did not significantly affect the milk yield per milking nor the major milk components. The concentrations of total Ca, P, Na, and K in the blood serum were not affected by dietary trace element intake, except for Mg, whose concentration increased in the supplemented animals. The milk yield per milking and the major milk components were significantly affected by the stage of lactation. The same factor affected the blood concentrations of all the investigated elements, which followed a decreasing trend during the experimental period, though remaining within the physiological range. These data are of interest both for clinical practice and research, as well as for the dairy donkey management to optimize animals’ health and productivity.

## Figures and Tables

**Table 1:** Milk yield and composition from donkeys supplemented (TE, n = 8) or not (CTL, n = 8) with dietary trace elements (mean of 6 sampling times).

	Treatments ^1^			
Item	CTL	TE	SEM ^2^	*p* Value
Milk yield per milking, mL	528	583	29.1	0.21
Protein, g/100 g	1.73	1.76	0.02	0.49
Fat, g/100 g	0.19	0.25	0.05	0.42
Lactose, g/100 g	7.09	7.06	0.03	0.59
Ash, g/100 g	0.40	0.39	0.01	0.36

^1^ Treatments: CTL = control diet provided Zn 34.1 mg/kg, Fe 254.9 mg/kg, Cu 7.5 mg/kg, Mn 42.1 mg/kg, Se 0.05 mg/kg, Co 0.17 mg/kg, and I 0.36 mg/kg (as fed); TE = trace-element-supplemented diet provided Zn 88.5 mg/kg, Fe 316.5 mg/kg, Cu 19.6 mg/kg, Mn 113.0 mg/kg, Se 0.27 mg/kg, Co 1.10 mg/kg, and I 1.42 mg/kg (as fed). ^2^ Standard error of mean.

**Table 2:** Total macro minerals concentrations in blood serum of lactating donkeys supplemented (TE, n = 8) or not (CTL, n = 8) with dietary trace elements (mean of 6 sampling times).

	Treatments ^1^			
Element	CTL	TE	SEM ^2^	*p* Value
Ca, mg/L	129.4	129.7	0.91	0.83
P, mg/L	105.0	102.8	3.33	0.67
Mg, mg/L	25.0	26.6	0.41	0.031
Na, mmol/L	137.2	137.0	0.48	0.80
K, mmol/L	4.62	4.67	0.05	0.49
Na to K ratio	29.6	29.5	0.43	0.87

^1^ Treatments: CTL = control diet provided Zn 34.1 mg/kg, Fe 254.9 mg/kg, Cu 7.5 mg/kg, Mn 42.1 mg/kg, Se 0.05 mg/kg, Co 0.17 mg/kg, and I 0.36 mg/kg (as fed); TE = trace-element-supplemented diet provided Zn 88.5 mg/kg, Fe 316.5 mg/kg, Cu 19.6 mg/kg, Mn 113.0 mg/kg, Se 0.27 mg/kg, Co 1.10 mg/kg, and I 1.42 mg/kg (as fed).^2^ Standard error of mean.

## Data Availability

The data are available from the authors upon request.
